# Prevalence of uterine rupture among women with one prior low transverse cesarean and women with unscarred uterus undergoing labor induction with PGE2: A systematic review and meta-analysis

**DOI:** 10.1371/journal.pone.0253957

**Published:** 2021-07-06

**Authors:** Giuseppe Chiossi, Roberto D’Amico, Anna L. Tramontano, Veronica Sampogna, Viola Laghi, Fabio Facchinetti

**Affiliations:** 1 Division of Obstetrics, Department of Medical and Surgical Sciences for Mother, Child and Adult, University of Modena and Reggio Emilia, Modena, Italy; 2 Statistics Unit, Department of Diagnostic and Clinical Medicine and Public Health, University of Modena and Reggio Emilia, Modena, Italy; University of Newcastle, UNITED STATES

## Abstract

**Background:**

As uterine rupture may affect as many as 11/1000 women with 1 prior cesarean birth and 5/10.000 women with unscarred uterus undergoing labor induction, we intended to estimate the prevalence of such rare outcome when PGE2 is used for cervical ripening and labor induction.

**Methods:**

We searched MEDLINE, ClinicalTrials.gov and the Cochrane library up to September 1^st^ 2020. Retrospective and prospective cohort studies, as well as randomized controlled trials (RCTs) on singleton viable pregnancies receiving PGE2 for cervical ripening and labor induction were reviewed. Prevalence of uterine rupture was meta-analyzed with Freeman-Tukey double arcsine transformation among women with 1 prior low transverse cesarean section and women with unscarred uterus.

**Results:**

We reviewed 956 full text articles to include 69 studies. The pooled prevalence rate of uterine rupture is estimated to range between 2 and 9 out of 1000 women with 1 prior low transverse cesarean (5/1000; 95%CI 2-9/1000, 122/9000). The prevalence of uterine rupture among women with unscarred uterus is extremely low, reaching at most 0.7/100.000 (<1/100.000.000; 95%CI <1/100.000.000–0.7/100.000, 8/17.684).

**Conclusions:**

Uterine rupture is a rare event during cervical ripening and labor induction with PGE2.

## Introduction

Approximately 1 in 5 women in the USA, UK, and Europe undergo labor induction [1, 2]. As the unripe cervix represents a major impediment to achieve vaginal delivery in case of induced labor, various pharmacological and mechanical methods were developed to achieve cervical ripening [3, 4]; among these, prostaglandin E2 (PGE2, or Dinoprostone) has been used since the 1960s as it induces both cervical maturation and uterine contractions [5].

Uterine rupture is a rare obstetric complication that continues to be associated with a high rate of perinatal and maternal morbidity and mortality [6, 7]. The main risk factor for uterine rupture is a scarred uterus, usually from a prior cesarean birth, while rupture of the unscarred uterus is a rare event. Due to the potential health advantages for both mothers and infants, induction of labor has become a valid alternative for women undergoing trial of labor after cesarean (TOLAC) [8], even if the risk of uterine rupture is higher when labor is induced as opposed to spontaneous [7, 9].

Studies on the relationship between cervical ripening agents and induction of labor are generally small, underpowered to detect relevant differences, and difficult to use for definitive conclusions [10]; furthermore, prostaglandins as a class have demonstrated inconsistent effects on the risk of uterine rupture [10]. While PGE2 is commonly used among women with unscarred uterus due to its safety profile [10], 3 major societies have adopted different policies when the ripening agent is prescribed to women with a prior cesarean birth. According to the 2008 guidelines from The National Institute for Health and Care Excellence women with a previous cesarean may be offered induction of labor with vaginal PGE2 [2]; instead, the Society of Obstetricians and Gynecologists of Canada has recommended against the use of PGE2 since 2005 due to the increased risk of uterine rupture, except in rare circumstances [11], while the American Congress of Obstetrics and Gynecology does not provide definitive recommendations on the use of PGE2 due to limited evidence, as indicated in a 2019 official statement [8].

Currently, the practice of labor induction continues to increase, PGE2 is widely used for cervical maturation, and concerns for the safety of mother and infant are prominent factors in decisions about childbirth, especially among at risk categories such as women with a history of cesarean birth; therefore, we conducted a systematic review and meta-analysis to determine the prevalence of uterine rupture among women with 1 prior low transverse cesarean and among those with an unscarred uterus receiving PGE2 for cervical ripening and labor induction at term.

## Materials and methods

We searched MEDLINE, ClinicalTrials.gov (www.clinicaltrials.gov), and the Cochrane Collaboration databases from inception up to September 1^st^ 2020, using a combination of text words including “induction of labor”, “induced labor”, “prostaglandin E2”, “dinoprostone”, “vaginal birth after cesarean”, “trial of labor after cesarean”, “prior cesarean”, and “uterine rupture” (S1 Table). Searches were not limited by geographic region or language. References of included studies were also hand-searched for additional eligible studies. Attempts were made to contact authors of the original manuscripts for additional information if required (eg. abstracts).

Studies were included if they specified the number of women receiving PGE2 for cervical ripening as well as the number of uterine rupture cases. The analysis only addressed complete uterine rupture, while instances of incomplete uterine tears were excluded. Uterine rupture was defined as a full thickness laceration of the uterine wall that also includes the overlying peritoneum, associated with fetal distress, the need for an emergency cesarean, uterine repair or hysterectomy, severe bleeding, or protrusion/expulsion of the placenta and/or fetus into the abdominal cavity [12]. Instead, incomplete rupture does not extend through the entire thickness of the uterus and overlying peritoneum, it usually presents less acutely without affecting the fetal status, and it is discovered incidentally (as during a repeat cesarean section, when the uterine scar stretches so thin that only the peritoneum seems to separate the uterine from the abdominal cavity; condition also known as uterine dehiscence, or uterine window) [12]. As uterine rupture does not go undetected due to the consequences on both maternal and fetal wellbeing, we considered as 0 the number of cases from those studies designed to investigate “maternal and/or neonatal safety” or “intrapartum maternal morbidity” even if they did not explicitly mention such rare complication. As only women receiving PGE2 for labor induction were studied, we excluded cases of rupture preceding the onset of labor, that occurred during spontaneous labor, or were attributed to trauma, such as the ones from obstetric interventions (rotational forceps, internal podalic version, or fundal pressure) or nonobstetric (violent) origin. Only singleton pregnancies at ≥ 37 weeks’ gestation, with a viable fetus prior to the onset of labor were included in the analysis, while second trimester terminations, preterm gestations, as well as cases of labor induction due to intrauterine fetal demise were excluded. Among women with scarred uterus, only the ones with one prior low transverse uterine incision were studied; subjects with a history of low vertical or classical uterine incisions, with more than 1 cesarean birth, or with other types of prior uterine surgery (i.e myomectomy) were excluded. We only analyzed data on primiparous women when surveys did not clearly specify if or which multipara had a prior cesarean birth.

We quantified the risk of uterine rupture as prevalence rates, comparing the number of mothers who experienced such complication with the total number of women receiving PGE2 for cervical ripening. We chose not to express uterine rupture as incidence rate, as authors did not consistently specify the time period during which each study population was observed. Only data from retrospective cohort studies, prospective cohort studies, and RCTs were included in the analysis, as case reports, and case control studies don’t describe the entire population at risk (i.e. total number of women exposed to PGE2). Pooled prevalence rates of uterine rupture were estimated using Freeman-Tukey double arcsine transformation, which stabilizes the variances of the included studies (metaprop_one, Stata software package version 15.1, College Station, Tx, USA). Between-study heterogeneity was accounted for using random-effects meta-analyses.

Two reviewers (GC and ALT) independently evaluated the titles and abstracts of all citations produced by the electronic searches. The full text of the potentially eligible studies was retrieved and independently assessed for eligibility by two review authors (GC and ALT). Discordance between reviewers was resolved by discussion, and if not a third author (FF) was involved. When multiple publications addressed the same cases, the main study report was used as reference. Data were extracted in a standardized manner by 3 authors (ALT, VL, and GC).

To better describe our study population, we investigated the different PGE2 pharmaceutical formulations utilized in the included studies, along with the induction agents PGE2 was compared to (if the study design required a comparator). Moreover, maternal characteristics (age, race, parity, BMI), obstetric variables (indication for labor induction, gestational age at labor induction, tachysystole/hyperstimulation rates, cesarean delivery rates), and neonatal features (birth weight, NICU admission) were reported. Tachysystole was defined as more than five contractions per 10 min [2, 13]. Uterine hyperstimulation was defined as a single contraction lasting at least 2 min, or more than five contractions over 20-minute period with adverse changes in the fetal heart rate pattern on cardiotocography [2].

Continuous variables presented as means in the original studies, were summarized as means of the means reported in the original publications. Categorical variables presented as proportions in the original studies, were summarized as means of the proportions specified in the original publications. All statistical analyses were performed with the Stata software package version 15.1 (StataCorp, College Station, TX). Dataset was included among the Supporting Information to the manuscript.

This systematic review was conducted according to the MOOSE (Meta-analysis of Observational Studies in Epidemiology) and PRISMA (Preferred Reporting Items for Systematic Reviews and Meta-Analyses) statements. Although no RCT intended to address our study question, information about uterine rupture was often provided when PGE2 effects were investigated with this study design; therefore, MOOSE reporting guidelines were followed even when prevalence of uterine rupture was inferred from RCTs [14]. A risk of bias tool for prevalence studies was considered to evaluate the quality of the publications included in the meta-analysis [15]. Our review was not registered, and it was exempt from IRB approval as it collected and integrated publicly available research.

## Results

**Fig 1:**
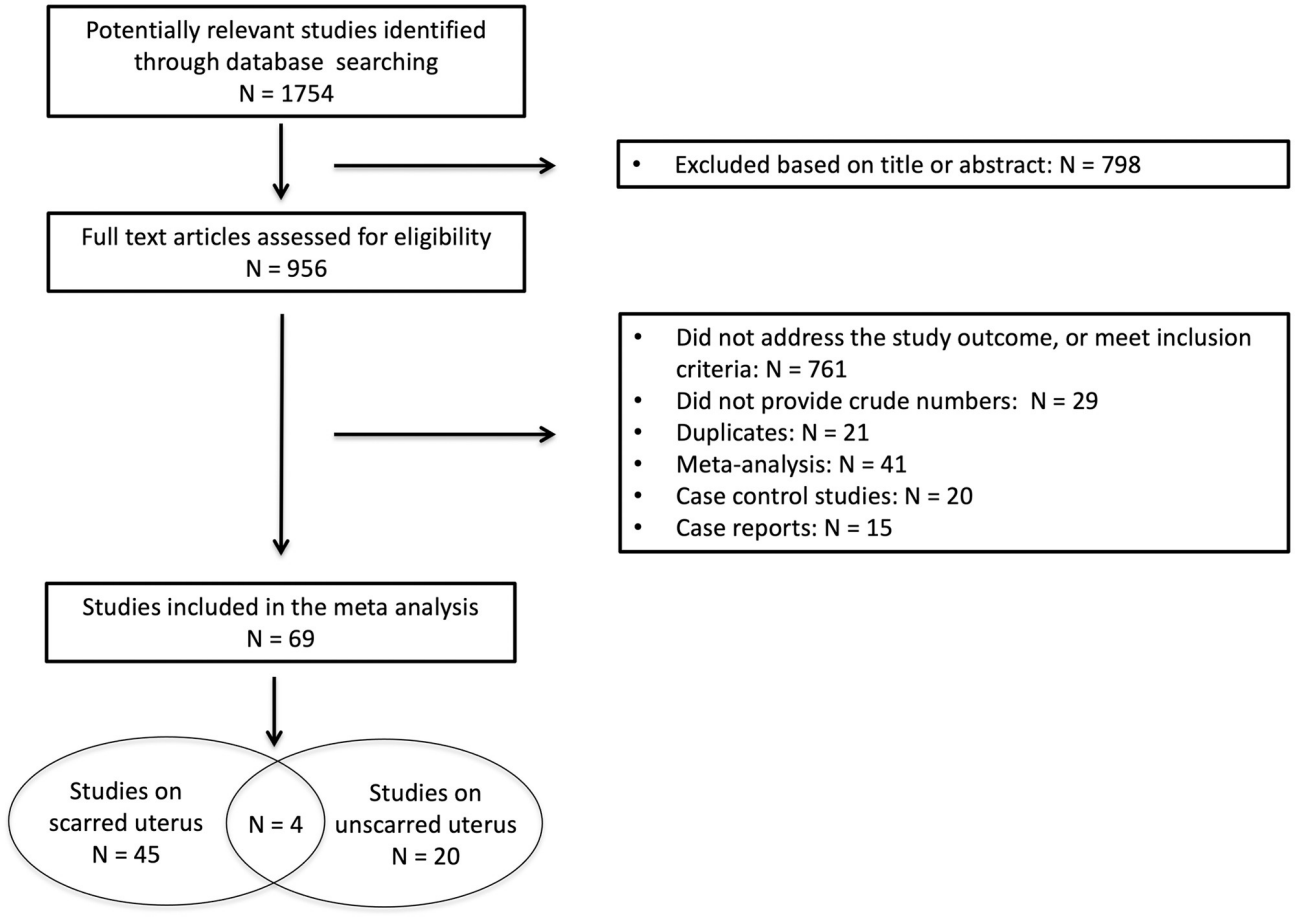
Flow diagram of the included studies.

The flow diagram of the electronic search details and selection process are shown in Fig 1. We identified 1754 publications and reviewed 956 full-length articles, to include 69 eligible studies (53 retrospective cohort studies, 4 prospective cohort studies, and 12 RCTs). Such publications specifically included uterine rupture among the study outcomes, with the exception of 4 studies designed to investigate “maternal and/or neonatal safety” [16–19] and 1 study focused on “intrapartum maternal morbidity” [20], where we considered as 0 the number of uterine rupture cases even if it was not explicitly mentioned. As uterine rupture does not go undetected due to the consequences on both maternal and fetal wellbeing, cases of such complication would be reported in publications addressing safety.

**Fig 2:**
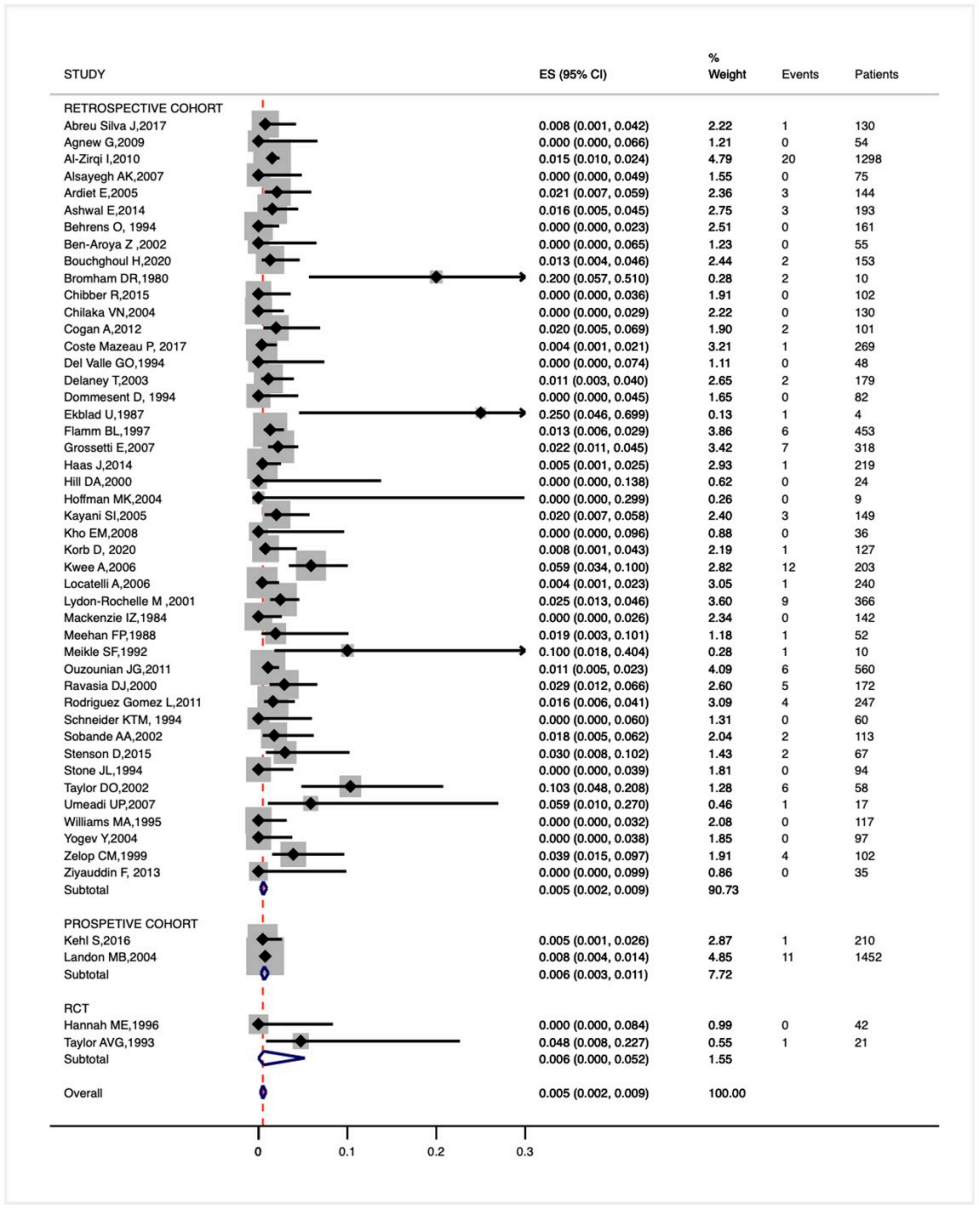
Forest plot for the prevalence of uterine rupture among women with 1 prior low transverse cesarean treated with PGE2.

The overall pooled prevalence rate of uterine rupture among women with 1 prior low transverse cesarean is 5/1000 (95%CI 2-9/1000, I^2^ 47.7%, 122/9000). Rates are similar across all study designs, being 5/1000 (95%CI 2-9/1000, 109/7275) according to retrospective cohort studies, 6/1000 (95%CI 3-11/1000, 12/1662) considering prospective cohort studies, and of 6/1000 (95%CI 0.1-52/1000, 1/63) including only RCTs (Fig 2).

**Fig 3:**
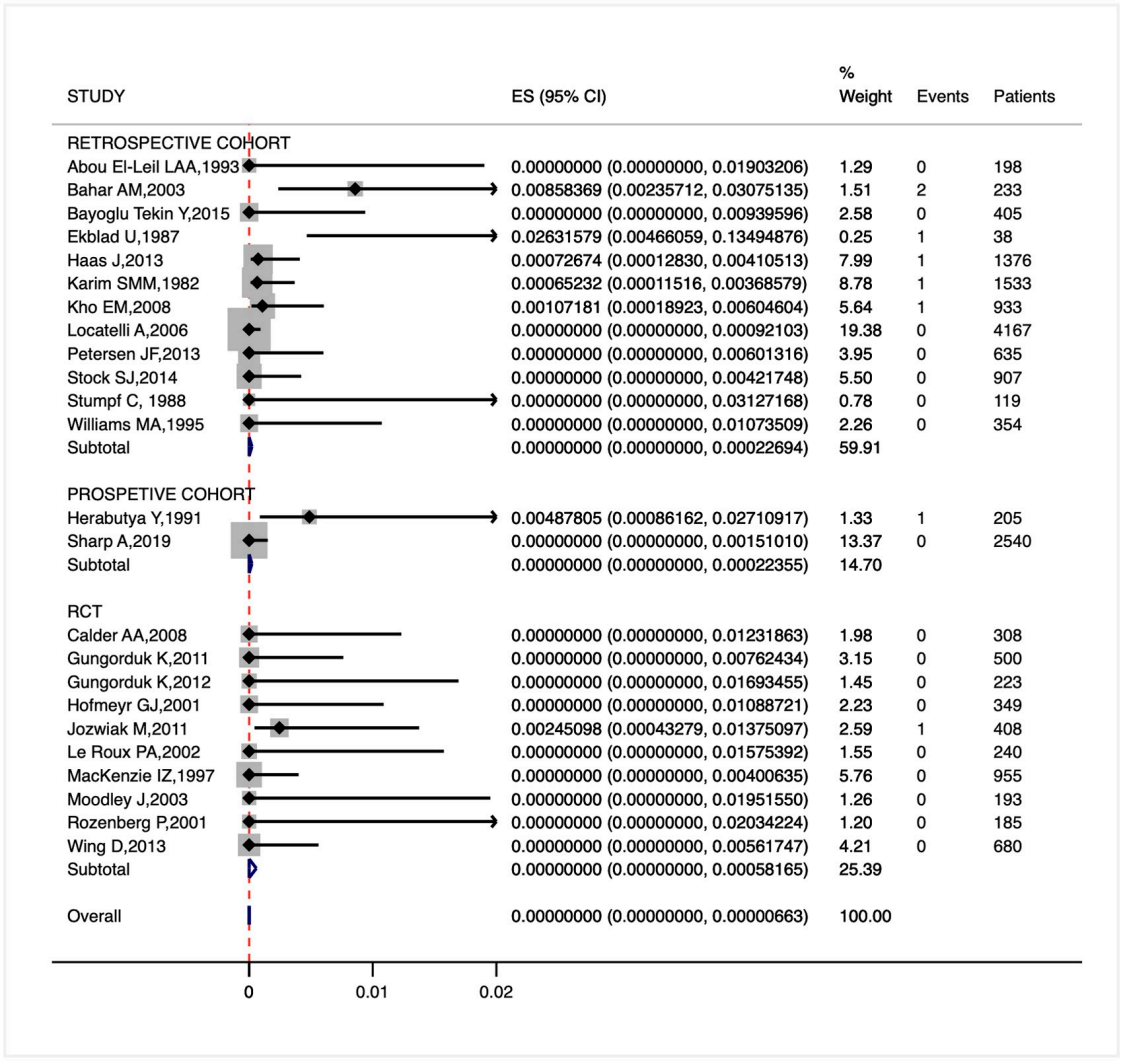
Forest plot for the prevalence of uterine rupture among women with unscarred uterus treated with PGE2.

The prevalence of uterine rupture among women with unscarred uterus is extremely low. Only 7 out of 24 studies included in our meta-analysis reported at most 2 cases of uterine rupture, totaling 8 instances (6 in retrospective cohort studies, 1 in a prospective cohort study, and 1 in a RCT) among 17.684 women receiving PGE2 for cervical ripening and labor induction (10.898 in retrospective cohort studies, 2745 in prospective cohort studies, and 4041 in RCTs). Fig 3 presents the prevalence of the outcome in each study and its synthesis: we are 95% confident that the overall pooled prevalence of uterine rupture is at most 0.7/100.000 among women with no prior low transverse cesarean section (I^2^ 0.5%), reaching the highest value of 22/100.000 according to retrospective cohort studies, 22/100.000 considering prospective cohort studies, and 58/100.000 analyzing RCTs only.

The characteristics of the entire study populations are shown in Table 1, while the features of each included study are presented in S2 Table. Of note, vaginal or intracervical gel was the most common PGE2 pharmaceutical formulation utilized for cervical ripening (75.5% among those with 1 prior CD, 78.2% among women with unscarred uterus), while oxytocin is the induction agent PGE2 was more frequently compared to among women with prior uterine surgery (39.1%), as opposed to PGE1 among women with unscarred uterus (47%).

**Table 1:** Characteristics of the study population.

Characteristics	Prevalence	Reference
**a) Studies on women with 1 prior low transverse cesarean section**		
PGE2 formulation		
Gel	34 (75.5%)	[7, 19, 21–52]
Pessary	7 (15.5%)	[53–59]
Tablet	1 (2.2%)	[60]
Multiple formulations	3 (6.8%)	[61–63]
Comparator		
Different PGE2 formulation	1 (4.4%)	[57]
PGE1	1 (4.4%)	[63]
Mechanical method	4 (17.4%)	[26, 31, 38, 39]
Multiple Agents	2 (8.6%)	[33, 58]
Oxytocin	9 (39.1%)	[7, 30, 32, 36, 40, 44, 51, 56, 59]
Placebo or expectant management	5 (21.7%)	[19, 22, 28, 52, 53]
Other: PGF, AROM, or IMN	1 (4.4%)	[48]
Maternal Age	30.9 (26–35.3)	[7, 19, 22, 24–28, 30–32, 35, 39, 41, 43, 45, 49, 51–53, 55–59, 62]
BMI (Kg/m^2^)	27.1 (25.7–29.1)	[7, 25, 27, 51, 55]
Non-Caucasian	47.4% (5.3–72%)	[30, 42, 46, 56, 57]
Indication for induction of labor		[19, 21, 22, 24, 27–29, 33, 35, 38, 42, 45, 46, 49, 51, 52, 57–59, 61, 62, 64]
Maternal	27% (0–64%)	
Fetal	15.2% (0–75.8%)	
Post dates	37.9% (0–86%)	
PROM	15.1% (0–100%)	
Other	13.4% (0–58.7%)	
Gestational age (weeks)	39.3 (38.3–40.2)	[7, 19, 22, 26, 28, 30, 31, 35, 41, 43–45, 49, 51–53, 56–59, 62, 64]
Augmentation	44.4% (10.6–88.3%)	[7, 19, 22, 24, 26–29, 32–34, 38, 41, 42, 44–46, 49–51, 57, 60, 61, 65]
Hyperstimulation/Tachysystole	7.2% (0–25%)	[19, 33, 42, 45, 57, 62, 64]
Cesarean birth	34.1% (3.9%– 76.5%)	[19, 21–30, 33–35, 37–39, 41, 42, 44–46, 48, 49, 51–53, 55, 57–59, 61, 62, 64, 66]
Birth-weight (g)	3308 (2981–3580)	[7, 19, 22, 24–26, 28, 30–33, 35, 41, 44, 45, 49–51, 53, 55, 56, 59]
Neonatal admission	8.6% (0.7%– 16%)	[19, 24, 30, 38, 46, 51, 53, 55, 57, 58, 62]
Publications in languages other than English	4 (8.1%)	[25, 55, 62, 66]
**b) Studies on women with unscarred uterus**		
PGE2 formulation		
Gel	18 (78.2%)	[17, 19, 20, 33, 41, 67–79]
Pessary	5 (21.8%)	[16, 57, 80–82]
Comparator		
Different PGE2 formulation	5 (29.4%)	[20, 57, 67, 68, 81]
PGE1	8 (47%)	[73, 75, 77–80, 82, 83]
Mechanical method	1 (5.9%)	[76]
Oxytocin	2 (11.8%)	[33, 81]
Placebo or expectant management	1 (5.9%)	[19]
Maternal Age	28.9 (25.9–34)	[16, 18, 19, 41, 57, 67–70, 73–78, 81, 82]
BMI (Kg/m^2^)	28.1 (24.8–34.1)	[18, 70, 74, 76, 81, 82]
Primiparous	47.5% (0–81.5%)	[17, 18, 20, 57, 67, 69, 71, 72, 74–78, 81–83]
Non-Caucasian	39.4% (17–56.3%)	[57, 76, 82]
Indication for induction of labor		[16–20, 33, 57, 67–79, 81, 83]
Maternal	22% (0–60%)	
Fetal	14.4% (0–40.5%)	
Post dates	37.1% (0–100%)	
PROM	9.6% (0–100%)	
Other	16.6% (0–40%)	
Gestational age (weeks)	39.8 (38–41.4)	[16, 19, 20, 41, 57, 67–71, 73–78, 82]
Augmentation	54.9% (16–100%)	[16, 19, 20, 33, 41, 57, 72–79, 81]
Hyperstimulation/Tachysystole	5.2% (0–25%)	[16, 18–20, 33, 57, 68, 70, 72–79, 81, 82]
Cesarean birth	22.8% (3.4%– 53.2%)	[16, 17, 19, 20, 33, 41, 57, 67–79, 81–83]
Birth-weight (g)	3388 (3100–3729)	[16, 19, 20, 33, 41, 68, 70, 72, 74, 79, 81]
Neonatal admission	6.1% (1%– 20.8%)	[16, 19, 20, 57, 70, 74, 76, 77, 79, 81–83]
Publications in languages other than English	1 (4.2%)	[71]

Prevalence: number (%) of studies addressing each characteristic

Reference: publications addressing each specific characteristic.

PGF: prostaglandin F, AROM: artificial rupture of membranes, IMN: isosorbide mononitrate

Continuous variables presented as means in the original studies (maternal age, gestational age, BMI, and birth-weight), are presented as means (min—max) of the means reported in the original publications. Categorical variables presented as proportions in the original studies (primiparity, race, indications for labor induction, augmentation, hyperstimulation/tachysystole, cesarean delivery, and neonatal admission) are presented as means (min—max) of the proportions specified in the original publications. Study-specific categorical variables (PGE2 formulation, and comparator) are presented as the number (%) of studies presenting such descriptors.

**Fig 4:**
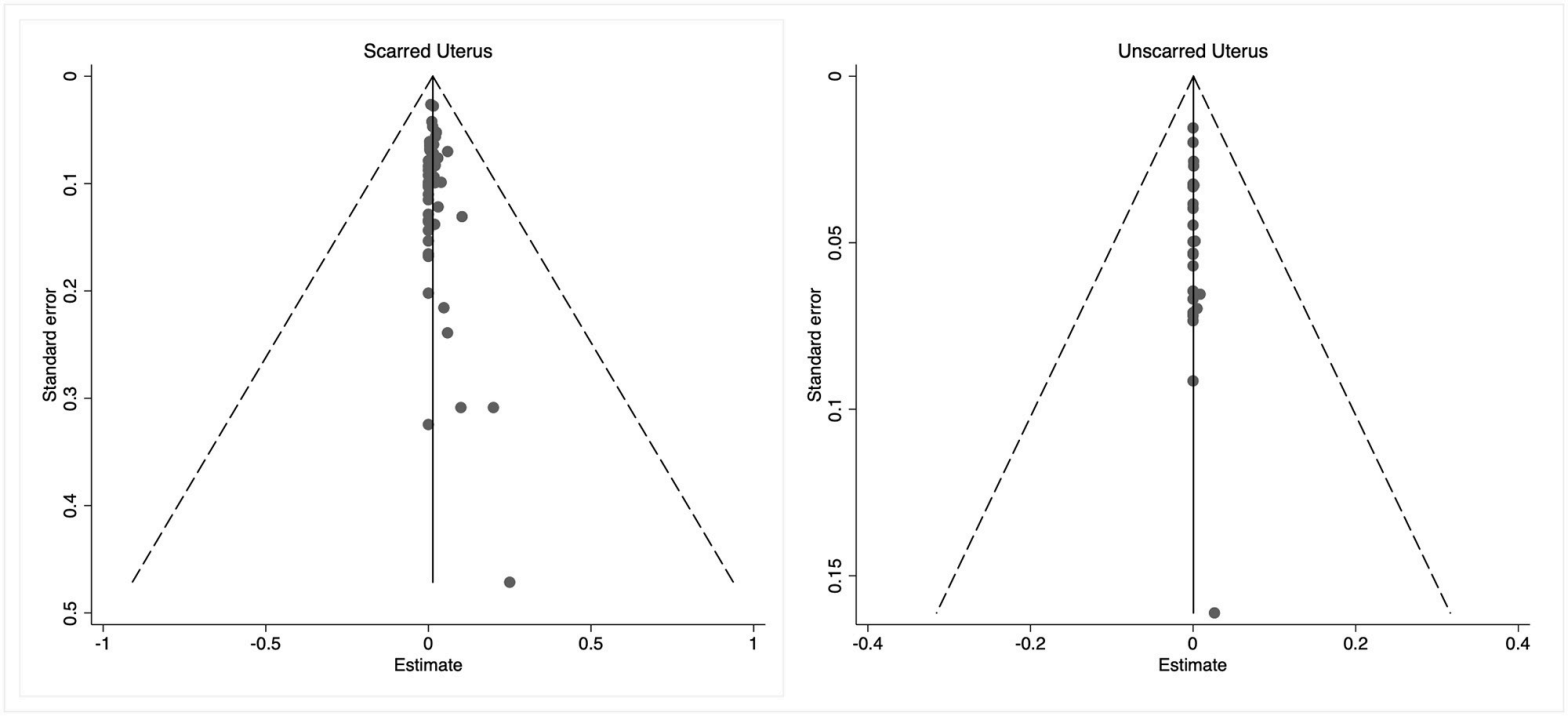
Publication bias of the included studies.

When confronted with the risk of bias tool for prevalence studies [15], all the included publications were considered as low risk, due to the specific nature of our study question, and how the outcome is measured: uterine rupture has overt clinical manifestations, and it is objectively documented by obstetricians at the time of cesarean birth (Table 2). No publication bias was found according to funnel plot (Fig 4).

**Table 2:** Quality assessment and risk of bias of the included studies.

	Was the study’s target population a close representation of the national population?	Was the sampling frame a true or close representation of the target population?	Was some form of random selection or a census used to select the sample?	Was the likelihood of non-response bias minimal?	Were data collected directly from the subjects (as opposed to a proxy)?	Was an acceptable case definition used in the study?	Was the study instrument that measured the parameter of interest shown to be reliable and valid?	Was the same mode of data collection used for all subjects?	Were the numerator(s) and denominator for the parameter of interest appropriate?	Summary on the overall risk of study bias	
**a) Studies on women with 1 prior low transverse cesarean section**											
Abreu Silva J, 2017	1	1	1	0	0	0	0	0	0	3	Low
Agnew G, 2009	1	1	1	0	0	0	0	0	0	3	Low
Al-Zirqui I, 2010	1	1	1	0	0	0	0	0	0	3	Low
Alsayegh AK, 2007	1	1	1	0	0	0	0	0	0	3	Low
Ardiet E, 2005	1	1	1	0	0	0	0	0	0	3	Low
Ashwal E, 2014	0	1	1	0	0	0	0	0	0	2	Low
Behrens O, 1994	1	1	1	0	0	0	0	0	0	3	Low
Ben-Aroya Z, 2002	1	1	1	0	0	0	0	0	0	3	Low
Bouchghoul H, 2020	1	1	1	0	0	0	0	0	0	3	Low
Bromham DR, 1980	1	1	1	0	0	0	0	0	0	3	Low
Chibber R, 2015	1	1	1	0	0	0	0	0	0	3	Low
Chilaka VN, 2004	1	1	1	0	0	0	0	0	0	3	Low
Cogan A, 2012	1	1	1	0	0	0	0	0	0	3	Low
Coste Mazeau P, 2017	1	1	1	0	0	0	0	0	0	3	Low
Del Valle GO, 1994	1	1	1	0	0	0	0	0	0	3	Low
Delaney T, 2003	1	1	1	0	0	0	0	0	0	3	Low
Dommesent D, 1994	1	1	1	0	0	0	0	0	0	3	Low
Flamm BL, 1997	1	1	1	0	0	0	0	0	0	3	Low
Grossetti E, 2007	1	1	1	0	0	0	0	0	0	3	Low
Haas J, 2014	1	1	1	0	0	0	0	0	0	3	Low
Hill DA, 2000	1	1	1	0	0	0	0	0	0	3	Low
Hoffman MK, 2004	1	1	1	0	0	0	0	0	0	3	Low
Kayani SI, 2005	1	1	1	0	0	0	0	0	0	3	Low
Korb D, 2020	1	1	1	0	0	0	0	0	0	3	Low
Kwee A, 2007	1	1	1	0	0	0	0	0	0	3	Low
Lydon Rochelle M, 2001	1	1	1	0	0	0	0	0	0	3	Low
Mackanzie IZ, 1984	1	1	1	0	0	0	0	0	0	3	Low
Meehan FP, 1988	1	1	1	0	0	0	0	0	0	3	Low
Meikle SF, 1992	1	1	1	0	0	0	0	0	0	3	Low
Ouzonian JG, 2011	1	1	1	0	0	0	0	0	0	3	Low
Ravaisa DJ, 2000	1	1	1	0	0	0	0	0	0	3	Low
Rodriguez Gomez L, 2011	1	1	1	0	0	0	0	0	0	3	Low
Schneider KTM, 1994	1	1	1	0	0	0	0	0	0	3	Low
Sobande AA, 2002	1	1	1	0	0	0	0	0	0	3	Low
Stenson D, 2015	1	1	1	0	0	0	0	0	0	3	Low
Stone JL, 1994	1	1	1	0	0	0	0	0	0	3	Low
Taylor DO, 2002	1	1	1	0	0	0	0	0	0	3	Low
Umeadi UP, 2007	1	1	1	0	0	0	0	0	0	3	Low
Yogev Y, 2004	1	1	1	0	0	0	0	0	0	3	Low
Zelop CM, 1999	1	1	1	0	0	0	0	0	0	3	Low
Ziyauddin F, 2013	1	1	1	0	0	0	0	0	0	3	Low
Kehl S, 2016	1	1	1	0	0	0	0	0	0	3	Low
Landon MB, 2004	0	1	1	0	0	0	0	0	0	2	Low
Hannah ME, 1996	1	1	0	0	0	0	0	0	0	2	Low
Taylor AVG, 1993	0	1	0	0	0	0	0	0	0	1	Low
**b) Studies on women with unscarred uterus**											
Abu El-Leil LAA, 1993	1	1	1	0	0	0	0	0	0	3	Low
Bahar AM, 2003	1	1	1	0	0	0	0	0	0	3	Low
Bayoglu Tekin Y, 2015	1	1	1	0	0	0	0	0	0	3	Low
Haas J, 2013	1	1	1	0	0	0	0	0	0	3	Low
Karim SMM, 1982	1	1	1	0	0	0	0	0	0	3	Low
Petersen JF, 2013	1	1	1	0	0	0	0	0	0	3	Low
Stock SJ, 2014	1	1	1	0	0	0	0	0	0	3	Low
Stumpf C, 1988	1	1	1	0	0	0	0	0	0	3	Low
Herabutya Y, 1991	1	1	1	0	0	0	0	0	0	3	Low
Sharp A, 2019	1	1	1	0	0	0	0	0	0	3	Low
Calder AA, 2008	0	1	0	0	0	0	0	0	0	1	Low
Gungorduk K, 2011	1	1	1	0	0	0	0	0	0	3	Low
Gungorduk K, 2012	1	1	1	0	0	0	0	0	0	3	Low
Hofmeyr GJ, 2001	0	1	0	0	0	0	0	0	0	1	Low
Jozwiak M, 2011	1	1	1	0	0	0	0	0	0	3	Low
Le Roux PA, 2002	1	1	1	0	0	0	0	0	0	3	Low
MacKenzie IZ, 1997	1	1	1	0	0	0	0	0	0	3	Low
Moodley J, 2003	1	1	1	0	0	0	0	0	0	3	Low
Rozenberg P, 2001	1	1	1	0	0	0	0	0	0	3	Low
Wing DA, 2013	1	1	1	0	0	0	0	0	0	3	Low
**c) Studies including women with 1 prior low transverse cesarean and women with unscarred uterus**											
Ekblad U, 1987	1	1	1	0	0	0	0	0	0	3	Low
Kho EM, 2008	1	1	1	0	0	0	0	0	0	3	Low
Locatelli A, 2006	1	1	1	0	0	0	0	0	0	3	Low
Williams MA, 1995	1	1	1	0	0	0	0	0	0	3	Low

The overall risk of bias was considered as low if the cumulative score was 0–3, moderate if the score was 4–6, high if the score was 7–9

## Discussion

As uterine rupture is a rare event when PGE2 is used for cervical ripening, its prevalence needs to be estimated in studies with large populations to limit publication bias. We estimated that the pooled prevalence rate of uterine rupture is 5/1000 among women undergoing TOLAC and at most 0.7/100.000 among those with an unscarred uterus.

Women with history of low transverse cesarean birth are at higher risk of uterine rupture, as reported by several studies showing a clinically determined uterine rupture rate after TOLAC of approximately 5–9/1000 [7, 9, 84–87], that increases up to 11/1000 when oxytocin is administered for labor induction [7, 9, 88]. Isolated reports suggested an increased risk of uterine rupture in patients undergoing TOLAC when PGE2 was administered [47, 88]; however, multiple meta-analyses showed no association between the prostaglandin and disruption of the uterine wall [89–91]. Our analysis suggests that cervical ripening with PGE2 has rates of such complication that do not differ from the historic rates reported for labor induction among women with 1 prior low transverse cesarean.

Cervical ripening protocols, that differ in terms of PGE2 dose, pharmaceutical formulation and timing of administration, contribute to determine the safety and the success of TOLAC together with many other maternal and obstetric characteristics, such as history of prior vaginal deliveries, gestational age, fetal weight, labor augmentation, maternal age and BMI. Such wide range of contributing factors and a variety of different study designs led us to use the random effects model, which accounted for study heterogeneity. The contribution of different PGE2 dosages, pharmacological formulations and timing of administration was not further explored with meta regression due to the insufficient number of studies; instead, the role of study design was addressed with subgroup analyses.

Spontaneous rupture of the unscarred uterus is an extremely uncommon event. No cases were reported by two observational studies respectively analyzing 21,998 and 30,874 primigravid labors [92, 93]. When obstetric populations included both primigravida and multipara, the prevalence ranged from 0.6/10.000 (95%CI: 0.01-3/10.000) [94] to 5/10.000 (95%CI 3-9/10.000) [6]. Similarly, our analyses show low estimates of such complication. A review of case reports of uterine rupture in primigravid women underlined the association with specific risk factors [95]. Besides prior uterine surgery, uterine rupture was reported in women with uterine anomalies secondary to diethylstilbestrol exposure, bicornuate uteri, and abnormal placentation. Maternal connective tissue disease, in particular Ehlers-Danlos syndrome, was also associated with uterine rupture. Labor induction with misoprostol, and labor augmentation with oxytocin were both linked to rupture of the unscarred uterus, whilst none of the reported cases received PGE2 for cervical ripening [95]. We identified similar risk factors when uterine rupture complicated cervical ripening with PGE2: 2 cases occurred during oxytocin augmentation (in one instance infusion was inadvertently started only 4 hours after PGE2 administration) [72, 76], 1 case was diagnosed at the time of cesarean section for obstructed labor [57], 1 patient was a grandmultiparous (≥ 5 deliveries) with labor inducted the day after she underwent appendectomy [69], 1 patient was diagnosed with uterine rupture at the time of laparotomy, when she was found to have placenta accreta, after failed attempts to manually remove the placenta [17]. Two patients received high (3 mg) PGE2 doses [68], while only 1 nulliparous woman experienced rupture after going into labor with one single dose of 0.5 mg intracervical gel [33]. Our analysis establishes no clear associations between PGE2 use and rupture of the unscarred uterus, as uterine rupture rates are not higher than the ones reported in the literature for women with no prior uterine surgeries. Moreover, obstetric characteristics (i.e. multiparity, oxytocin infusion, cephalo-pelvic disproportion, abnormal placentation) that were identified as risk factors for disruption of the uterine wall, were also noted when such complication followed cervical ripening and labor induction with PGE2.

When PGE2 is administered for cervical ripening, we found a cesarean birth rate of 34.1% among women with 1 prior CD, and 22.8% in case of unscarred uterus (Table 1). Our findings are similar to the reported trends, as cesarean birth rates reached 32% among women with a scarred uterus undergoing labor induction/augmentation [96], 26% among women attempting TOLAC [1] and 18.6% among nulliparous women undergoing elective induction at term [97]. On average 7.2% of the mothers with 1 prior CD and 5.2% of the ones with unscarred uterus experienced hyperstimulation/tachysystole after PGE2 administration (Table 1): such high rates could be due to the report of episodes not associated with fetal heart rate changes, the inclusion of studies with different quality, and the lack of information from more than half of the included publications. The rate of NICU admissions is higher than the reported trends [98] (Table 1): as our population consisted in women undergoing induction of labor for maternal or fetal indications, it is possible that newborns were more likely to require higher level of care than children from low risk pregnancies.

Strengths of our study include the comprehensive search strategy with no language restrictions, as well as the low risk of bias of the included surveys. Limitations primarily relate to the underlying data that was available on this topic. Primary studies were not specifically designed to address our study question, and they were mostly observational in nature. As labor induction is a complex multi-step process, it becomes challenging to single out the role played by PGE2; moreover, a high degree of heterogeneity characterized the studies including women with 1 prior low transverse cesarean. Subgroup analyses on PGE2 formulation (gel, pessary, or tablet), dose, and route of administration (cervical, vaginal, or oral) were not performed due to the rarity of the outcome and the heterogeneity in study designs. For the same reasons, the potential additional risk from oxytocin infusion could not be addressed.

Despite our findings may reassure clinicians on cervical ripening with PGE2, a multicenter prospective cohort study or national registry would offer the best opportunity to assess the safety of such agent, especially among women with a prior low transverse cesarean. Different PGE2 dosages and administration protocols could be compared to other induction strategies, while accounting for confounding. In the meantime, the use of PGE2 should continue in case of no prior uterine surgery, and it should be considered among women undergoing TOLAC who are comfortable with the increased risks of uterine rupture associated with induction of labor.

## Supporting information

S1 Checklist PRISMA 2020 checklist. (DOCX)

S1 Dataset (DTA)

S2 Dataset (DTA)

S1 File MOOSE guidelines for meta-analyses and systematic reviews of observational studies*. (PDF)

S1 Table Electronic search. (DOCX)

S2 Table Characteristics of the included studies. (DOCX)
