# Supplementary material for: Prevalence of uterine rupture among women with one prior low transverse cesarean and women with unscarred uterus undergoing labor induction with PGE2: A systematic review and meta-analysis
Source: PLoS One. 2021 Jul 6;16(7):e0253957. doi: 10.1371/journal.pone.0253957 (PMC8259955; doi:10.1371/journal.pone.0253957)
Supplement: S1 File — (PDF) [file pone.0253957.s004.pdf]

MOOSE Guidelines for Meta-Analyses and Systematic Reviews of Observational Studies*

	Topic	Page numbe
Title	Identify the study as a meta-analysis (or systematic review)	1
Abstract	Use the journal's structured format	2
Introduction	**Present:**	
	The clinical problem	3
	The hypothesis	3-4
	A statement of objectives that includes the study population, the condition of interest, the exposure or intervention, and the outcome(s) considered	3-4
Sources	**Describe:**	
	Qualifications of searchers (eg, librarians and investigators)	4
	Search strategy, including time period included in the synthesis and keywords	4
	Effort to include all available studies, including contact with authors	4
	Databases and registries searched	4
	Search software used, name and version, including special features used (e.g. explosion)	4
	Use of hand searching (e.g, reference lists of obtained articles)	4
	List of citations located and those excluded, including justification	4
	Method of addressing articles published in languages other than English	4
	Method of handling abstracts and unpublished studies	4
	Description of any contact with authors	4
Study Selection	**Describe**	
	Types of study designs considered	6
	Relevance or appropriateness of studies gathered for assessing the hypothesis to be tested	6
	Rationale for the selection and coding of data (eg, sound clinical principles or convenience)	6
	Documentation of how data were classified and coded (eg, multiple raters, blinding, and inter-rater reliability)	6
	Assessment of confounding (e.g. comparability of cases and controls in studies where appropriate)	6
	Assessment of study quality, including blinding of quality assessors; stratification or regression on possible predictors of study results	7
	Assessment of heterogeneity	7
	Statistical methods (eg, complete description of fixed or random effects models, justification of whether the chosen models account for predictors of study results, dose-response models, or cumulative meta-analysis) in sufficient detail to be replicated	6-7
Results	**Present**	
	A graph summarizing individual study estimates and the overall estimate	fig 2, 3
	A table giving descriptive information for each included study	Table 1, 2
	Results of sensitivity testing (eg, subgroup analysis)	8, 9
	Indication of statistical uncertainty of findings	9
Discussion	**Discuss**	
	Strengths and weaknesses	18, 19
	Potential biases in the review process (eg, publication bias)	14

	Assessment of quality of included studies	14
	Consideration of alternative explanations for observed results	19
	Generalization of the conclusions (ie, appropriate for the data presented and within the domain of the literature review)	19
	Guidelines for future research	19
	Disclosure of funding source	1

*Modified from Stroup DF, Berlin JA, Morton SC, Olkin I, Williamson GD, Rennie D, et al. Meta-analysis of observational studies in epidemiology: a proposal for reporting. Meta-analysis Of Observational Studies in Epidemiology (MOOSE) group. JAMA 2000;283:2008–12. Copyrighted © 2000, American Medical Association. All rights reserved.
