# Supplementary material for: Prevalence of uterine rupture among women with one prior low transverse cesarean and women with unscarred uterus undergoing labor induction with PGE2: A systematic review and meta-analysis
Source: PLoS One. 2021 Jul 6;16(7):e0253957. doi: 10.1371/journal.pone.0253957 (PMC8259955; doi:10.1371/journal.pone.0253957)
Supplement: S1 Table — (DOCX) [file pone.0253957.s005.docx]

**S1 Table. Electronic search**

| **Search** | | **Number of publications** |
| --- | --- | --- |
| 1 | **(((dinoprostone) OR prostaglandin e2)) AND ((induced labor) OR labor induction)** Filters: **from 1000/1/1 - 2020/9/1** | 1702 |
| 2 | **(uterine rupture) AND ((prostaglandin e2) OR (dinoprostone)) Filters: from 1000/1/1 - 2020/9/1** | 207 |
| 3 | **((uterine rupture) AND ((prostaglandin e2) OR (dinoprostone)) AND (1000/1/1:2020/9/1[pdat])) NOT ((((dinoprostone) OR prostaglandin e2)) AND ((induced labor) OR labor induction) AND (1000/1/1:2020/9/1[pdat]))** | 52 |

Included manuscripts: search 1 + search 3= 1702 + 52= 1754
