# Supplementary material for: Prevalence of uterine rupture among women with one prior low transverse cesarean and women with unscarred uterus undergoing labor induction with PGE2: A systematic review and meta-analysis
Source: PLoS One. 2021 Jul 6;16(7):e0253957. doi: 10.1371/journal.pone.0253957 (PMC8259955; doi:10.1371/journal.pone.0253957)
Supplement: S2 Table — (DOCX) [file pone.0253957.s006.docx]

**S2 Table. Characteristics of the included studies.**

| **Study** | **Study Design** | **Unscarred uterus** | | **Scarred uterus** | | **PGE2 preparation** | **PGE2**  **Comparator** | **Age** | **BMI** | **Nullip (%)** | **Non Caucasian (%)** | **Indication for labor induction** | | | | | **Gest age (weeks)** | **Augment (%)** | **Hyperstim/**  **Tachysystole (%)** | **CD (%)** | **Birthweight (g)** | **NICU Admission (%)** | **Language** |
| --- | --- | --- | --- | --- | --- | --- | --- | --- | --- | --- | --- | --- | --- | --- | --- | --- | --- | --- | --- | --- | --- | --- | --- |
|  |  | **Cases (N)** | **Events (N)** | **Cases**  **(N)** | **Events (N)** |  |  |  |  |  |  | **Mat (%)** | **Fetal (%)** | **Post dates (%)** | **PROM (%)** | **Other (%)** |  |  |  |  |  |  |  |
| **Abou El-Leil LAA,1993** | Retrospective Cohort | 198 | 0 |  |  | Gel | Other PGE2 | 28 |  | 29 |  | 60 |  |  |  | 40 | 40 |  |  | 10 |  |  | English |
| **Abreu Silva J,2017** | Retrospective Cohort |  |  | 130 | 1 | Pessary | Placebo/Exp | 32 |  |  |  |  |  |  |  |  | 40 |  |  | 67 | 3400 | 7 | English |
| **Agnew G,2009** | Retrospective Cohort |  |  | 54 | 0 | Gel |  |  |  |  |  | 15 | 5 | 52 |  | 28 |  |  |  | 26 |  |  | English |
| **Al-Zirqi I,2010** | Retrospective Cohort |  |  | 1298 | 20 | Tablet |  |  |  |  |  |  |  |  |  |  |  | 15 |  |  |  |  | English |
| **Alsayegh AK,2007** | Retrospective Cohort |  |  | 75 | 0 | Gel | Placebo/Exp | 30.9 |  |  |  | 16 | 7 | 75 | 3 |  | 39 | 25 |  | 24 | 3163 |  | English |
| **Ardiet E,2005** | Retrospective Cohort |  |  | 144 | 3 | Gel |  |  |  |  |  |  |  |  |  |  |  |  |  | 36 |  |  | English |
| **Ashwal E,2014** | Retrospective Cohort |  |  | 193 | 3 | Gel |  | 35.3 |  |  |  | 9 | 29 | 22 | 20 | 20 |  | 49 |  | 16 | 3201 | 13 | English |
| **Bahar AM,2003** | Retrospective Cohort | 233 | 2 |  |  | Gel | Other PGE2 | 30.8 |  |  |  | 21 | 8 | 51 |  | 21 | 40 |  | 1 | 10 | 3100 |  | English |
| **Bayoglu Tekin Y,2015** | Retrospective Cohort | 405 | 0 |  |  | Pessary |  | 27.7 |  |  |  | 8 | 41 | 52 |  |  | 40 | 24 | 8 | 17 | 3300 | 2 | English |
| **Behrens O, 1994** | Retrospective Cohort |  |  | 161 | 0 | Gel |  | 29 | 26 |  |  | 27 |  | 30 | 34 | 9 |  |  |  | 25 | 3400 |  | German |
| **Ben-Aroya Z ,2002** | Retrospective Cohort |  |  | 55 | 0 | Gel | Mech | 29.3 |  |  |  |  |  |  |  |  | 39 | 16 |  | 46 | 3200 |  | English |
| **Bouchghoul H,2020** | Retrospective Cohort |  |  | 153 | 2 | Gel |  | 32 | 26 | 70 |  | 32 | 13 | 29 | 22 | 3 |  | 62 |  | 44 |  |  | English |
| **Bromham DR,1980** | Retrospective Cohort |  |  | 10 | 2 | Pessary |  |  |  |  |  |  |  |  |  |  |  |  |  |  |  |  | English |
| **Calder AA,2008** | RCT | 308 | 0 |  |  | Gel | PGE1 | 29.7 |  |  |  | 8 | 3 | 70 |  | 20 | 41 | 49 | 8 | 22 |  |  | English |
| **Chibber R,2015** | Retrospective Cohort |  |  | 102 | 0 | Gel | Placebo/Exp | 34.1 |  |  |  | 48 | 3 | 49 |  |  | 39 | 21 |  | 20 | 3300 |  | English |
| **Chilaka VN,2004** | Retrospective Cohort |  |  | 130 | 0 | Gel |  |  |  |  |  | 20 | 18 | 43 | 6 | 13 |  | 27 |  | 39 |  |  | English |
| **Cogan A,2012** | Retrospective Cohort |  |  | 101 | 2 | Gel | Multiple | 33 |  | 53 | 69 |  |  |  |  |  | 39 |  |  | 25 | 3200 | 12 | English |
| **Coste Mazeau P, 2017** | Retrospective Cohort |  |  | 269 | 1 | Pessary |  | 32 | 28 |  |  | 48 | 13 | 2 | 26 | 11 |  |  |  | 38 | 3211 | 9 | French |
| **Del Valle GO,1994** | Retrospective Cohort |  |  | 48 | 0 | Gel | Mech | 27.5 |  |  |  |  |  |  |  |  | 39 |  |  |  | 3200 |  | English |
| **Delaney T,2003** | Retrospective Cohort |  |  | 179 | 2 | Gel | Multiple | 29.2 |  |  |  |  |  |  |  |  |  | 48 |  |  | 3500 |  | English |
| **Dommesent D, 1994** | Retrospective Cohort |  |  | 82 | 0 |  |  |  |  |  |  | 12 | 24 | 41 | 12 | 11 |  |  |  | 23 |  |  | French |
| **Ekblad U,1987** | Retrospective Cohort | 38 | 1 | 4 | 1 | Gel | Oxytocin |  |  |  |  | 38 | 29 | 33 |  |  |  | 37 | 25 | 33 | 3400 |  | English |
| **Flamm BL,1997** | Retrospective Cohort |  |  | 453 | 6 | Gel |  |  |  |  |  |  |  |  |  |  |  | 77 |  | 49 |  |  | English |
| **Grossetti E,2007** | Retrospective Cohort |  |  | 318 | 7 | Pessary | Multiple | 31 |  | 32 | 5 |  |  |  |  |  | 39 |  |  |  | 3300 |  | English |
| **Gungorduk K,2011** | RCT | 500 | 0 |  |  | Pessary | Other PGE2 | 26.7 | 29 | 50 |  | 15 | 31 | 38 | 12 | 4 |  | 100 | 12 | 12 | 3175 | 5 | English |
| **Gungorduk K,2012** | RCT | 223 | 0 |  |  | Gel | Oxytocin | 26.6 | 30 | 55 |  |  |  |  | 100 |  | 39 | 100 | 8 | 18 | 3475 | 3 | English |
| **Haas J,2013** | Retrospective Cohort | 1376 | 1 |  |  | Gel |  | 34 |  |  |  | 11 | 35 | 17 | 17 | 21 | 40 |  |  | 3 |  |  | English |
| **Haas J,2014** | Retrospective Cohort |  |  | 219 | 1 | Gel |  | 34.1 |  |  |  | 20 | 37 | 15 | 13 | 16 | 40 |  |  | 4 | 3451 |  | English |
| **Hannah ME,1996** | RCT |  |  | 42 | 0 | Gel | Placebo/Exp | 28.5 |  | 60 |  |  |  |  | 100 |  | 39 |  |  | 40 |  |  | English |
| **Herabutya Y,1991** | Prospective Cohort | 205 | 1 |  |  | Gel |  |  |  | 82 |  | 22 | 9 | 64 |  | 4 |  | 72 | 1 | 41 | 3139 |  | English |
| **Hill DA,2000** | Retrospective Cohort |  |  | 24 | 0 | Gel | Multiple |  |  |  |  |  |  |  |  |  |  |  |  |  |  |  | English |
| **Hoffman MK,2004** | Retrospective Cohort |  |  | 9 | 0 | Gel |  |  |  |  |  |  |  |  |  |  |  |  |  | 33 |  |  | English |
| **Hofmeyr GJ,2001** | RCT | 349 | 0 |  |  | Gel | PGE1 | 27.2 |  | 47 |  | 14 | 9 | 43 | 18 | 16 | 40 | 33 | 8 | 17 |  |  | English |
| **Jozwiak M,2011** | RCT | 408 | 1 |  |  | Gel | Mech | 30.6 | 25 | 65 | 17 | 11 | 35 | 35 |  | 19 | 40 | 59 | 3 | 20 |  | 21 | English |
| **Karim SMM,1982** | Retrospective Cohort | 1533 | 1 |  |  | Gel |  |  |  | 36 |  | 44 | 13 | 33 |  | 11 |  |  |  | 6 |  |  | English |
| **Kayani SI,2005** | Retrospective Cohort |  |  | 149 | 3 | Gel | Mech |  |  |  |  | 26 | 14 | 44 | 10 | 6 |  | 34 |  | 44 |  | 1 | English |
| **Kehl S,2016** | Prospective Cohort |  |  | 210 | 1 | Gel | Multiple | 33.7 | 29 |  |  | 45 | 15 | 26 |  | 14 | 40 | 65 |  | 39 | 3542 | 16 | English |
| **Kho EM,2008** | Retrospective Cohort | 933 | 1 | 36 | 0 | Pessary | Other PGE2 | 33 |  | 61 | 45 | 31 | 14 | 35 |  | 20 | 40 | 55 | 3 | 30 |  | 5 | English |
| **Korb D, 2020** | Retrospective Cohort |  |  | 127 | 1 | Gel | Mech | 32.5 |  |  |  | 64 | 14 | 22 |  |  |  |  |  | 43 |  |  | English |
| **Kwee A,2006** | Retrospective Cohort |  |  | 203 | 12 | Gel | Multiple |  |  |  |  |  |  |  |  |  |  |  |  |  |  |  | English |
| **Landon MB,2004** | Prospective Cohort |  |  | 1452 | 11 | Gel | Multiple | 27 | 27 |  |  |  |  |  |  |  | 40 | 11 |  |  | 3360 |  | English |
| **Le Roux PA,2002** | RCT | 240 | 0 |  |  | Gel | PGE1 | 27.6 |  | 42 |  | 50 | 4 | 21 | 14 | 11 | 39 | 16 | 1 | 34 |  | 3 | English |
| **Locatelli A,2006** | Retrospective Cohort | 4167 | 0 | 240 | 1 | Gel |  | 30.9 |  |  |  |  |  |  |  |  | 39 | 42 |  | 15 | 3213 |  | English |
| **Lydon-Rochelle M ,2001** | Retrospective Cohort |  |  | 366 | 9 |  |  |  |  |  |  |  |  |  |  |  |  |  |  |  |  |  | English |
| **MacKenzie IZ,1997** | RCT | 955 | 0 |  |  | Gel | Other PGE2 |  |  | 52 |  | 16 | 6 | 68 |  | 10 | 41 | 41 | 0 | 8 | 3572 | 3 | English |
| **Mackenzie IZ,1984** | Retrospective Cohort |  |  | 142 | 0 | Multiple |  |  |  |  |  | 18 | 5 | 19 |  | 59 |  | 66 |  | 25 |  |  | English |
| **Meehan FP,1988** | Retrospective Cohort |  |  | 52 | 1 |  |  |  |  |  |  |  |  |  |  |  |  | 44 |  |  |  |  | English |
| **Meikle SF,1992** | Retrospective Cohort |  |  | 10 | 1 | Gel |  |  |  |  | 72 |  |  |  | 100 |  |  | 64 | 2 | 12 |  |  | English |
| **Moodley J,2003** | RCT | 193 | 0 |  |  | Gel | PGE1 | 26 |  | 48 |  | 19 | 34 | 8 | 4 | 35 | 38 | 31 | 9 | 42 |  |  | English |
| **Ouzounian JG,2011** | Retrospective Cohort |  |  | 560 | 6 | Gel |  | 30 |  |  |  |  |  |  |  |  | 40 |  |  |  |  |  | English |
| **Petersen JF,2013** | Retrospective Cohort | 635 | 0 |  |  |  | PGE1 |  |  | 49 |  | 15 | 12 | 41 |  | 33 |  |  |  | 21 |  | 8 | English |
| **Ravasia DJ,2000** | Retrospective Cohort |  |  | 172 | 5 | Gel | Multiple |  |  |  |  |  |  |  |  |  | 38 | 45 |  | 40 | 3188 |  | English |
| **Rodriguez Gomez L,2011** | Retrospective Cohort |  |  | 247 | 4 | Pessary | Oxytocin | 34 |  |  |  | 45 | 33 | 11 | 11 |  | 39 |  |  | 36 |  | 4 | English |
| **Rozenberg P,2001** | RCT | 185 | 0 |  |  | Gel | PGE1 |  |  |  |  | 27 | 2 | 68 |  | 3 |  | 81 | 3 | 25 | 3641 | 5 | English |
| **Schneider KTM, 1994** | Retrospective Cohort |  |  | 60 | 0 | Multiple |  | 31.7 |  |  |  | 48 | 9 |  | 24 | 19 | 39 |  | 11 | 32 |  | 8 | German |
| **Sharp A,2019** | Prospective Cohort | 2540 | 0 |  |  | Pessary | PGE1 | 29 | 26 | 47 |  | 15 | 29 | 11 | 11 | 34 |  |  | 0 |  |  |  | English |
| **Sobande AA,2002** | Retrospective Cohort |  |  | 113 | 2 | Gel |  | 29.3 |  |  |  | 5 | 9 | 29 | 15 | 42 | 39 | 21 | 0 | 45 | 2981 |  | English |
| **Stenson D,2015** | Retrospective Cohort |  |  | 67 | 2 | Multiple | PGE1 |  |  |  |  |  |  |  |  |  |  |  |  |  |  |  | English |
| **Stock SJ,2014** | Retrospective Cohort | 907 | 0 |  |  | Gel |  | 28.7 | 26 |  |  |  |  | 100 |  |  | 41 |  | 3 | 53 | 3729 | 1 | English |
| **Stone JL,1994** | Retrospective Cohort |  |  | 94 | 0 | Gel |  |  |  |  | 45 | 24 | 76 |  |  |  |  | 88 |  | 31 |  | 13 | English |
| **Stumpf C, 1988** | Retrospective Cohort | 119 | 0 |  |  | Gel |  |  |  | 33 |  | 33 |  |  | 30 | 37 | 40 |  |  | 24 |  |  | German |
| **Taylor AVG,1993** | RCT |  |  | 21 | 1 | Pessary | Multiple | 29.7 |  |  |  | 14 |  | 86 |  |  | 40 |  |  | 29 | 3300 |  | English |
| **Taylor DO,2002** | Retrospective Cohort |  |  | 58 | 6 | Gel |  |  |  |  |  |  |  |  |  |  |  |  |  |  |  |  | English |
| **Umeadi UP,2007** | Retrospective Cohort |  |  | 17 | 1 | Gel | Other |  |  |  |  |  |  |  |  |  |  |  |  | 77 |  |  | English |
| **Williams MA,1995** | Retrospective Cohort | 354 | 0 | 117 | 0 | Gel | Placebo/Exp | 29.9 |  |  |  | 28 | 8 | 29 | 6 | 29 | 39 | 84 | 2 | 36 | 3527 | 8 | English |
| **Wing D,2013** | RCT | 680 | 0 |  |  | Pessary | PGE1 | 25.9 | 34 | 66 | 56 |  |  |  |  |  | 40 |  | 0 | 27 |  | 10 | English |
| **Yogev Y,2004** | Retrospective Cohort |  |  | 97 | 0 | Gel |  | 30.9 |  |  |  | 30 | 24 | 16 | 11 | 19 | 39 | 25 |  | 36 | 3163 |  | English |
| **Zelop CM,1999** | Retrospective Cohort |  |  | 102 | 4 | Gel |  |  |  |  |  |  |  |  |  |  |  | 45 |  |  | 3580 |  | English |
| **Ziyauddin F, 2013** | Retrospective Cohort |  |  | 35 | 0 |  |  | 26 |  |  |  | 41 | 14 | 20 |  | 25 | 39 |  | 8 | 40 |  |  | English |

Cases: number of women undergoing labor induction; Events: number of documented uterine ruptures; Nullip: nulliparous; Mat: maternal, PROM: pre labor rupture of membranes; Gest age: gestational age; CD: cesarean delivery; Augment: augmentation; Hyperstim/Tachysystole: hyperstimulation or tachysystole; Placebo/exp: placebo or expectant management; Mech: mechanical methods; Other (Comparator): Prostaglandin F, artificial rupture of membranes, or isosorbide moninitrate
